# Supplementary material for: Relationship Between Alcohol Consumption and Vascular Structure and Arterial Stiffness in Adults Diagnosed with Persistent COVID: BioICOPER Study
Source: Nutrients. 2025 Feb 16;17(4):703. doi: 10.3390/nu17040703 (PMC11858638; doi:10.3390/nu17040703)
Supplement: Supplementary file 1 [file nutrients-17-00703-s001.zip › nutrients-3456054-supplementary.pdf]

nutrients

MDPI

Supplementary Materials

Relationship Between Alcohol Consumption and Vascular Structure and Arterial Stiffness in Adults Diagnosed with Persistent COVID: BioICOPER Study

Silvia Arroyo-Romero [1,2,†], Leticia Gómez-Sánchez [3,†], Nuria Suárez-Moreno [1,2], Alicia Navarro-Cáceres [1,2], Andrea Domínguez-Martín [1,2], Cristina Lugones-Sánchez [1,2,4], Olaya Tamayo-Morales [1,2,4], Susana González-Sánchez [1,4], Ana B. Castro-Rivero [1,2], Marta Gómez-Sánchez [5], Emiliano Rodríguez-Sánchez [1,2,4,6], Luis García-Ortiz [1,2,4,7], Elena Navarro-Matías [1,2,4,‡] and Manuel A. Gómez-Marcos [1,2,4,6,*,‡]

[1] Primary Care Research Unit of Salamanca (APISAL), Salamanca Primary Care Management, Institute of Biomedical Research of Salamanca (IBSAL), 37005 Salamanca, Spain; silvia_ar@usal.es (S.A.-R.); nuria.suarez@usal.es (N.S.-M.); alicia.nav@usal.es (A.N.-C.); andreadm@usal.es (A.D.-M.); crislugsa@gmail.com (C.L.-S.); olayatm@usal.es (O.T.-M.); gongar04@gmail.com (S.G.-S.); anacasri@yahoo.es (A.B.C.-R.); emiliano@usal.es (E.R.-S.); lgarciao@usal.es (L.G.-O.); enavarro@saludcastillayleon.es (E.N.-M.)

[2] Castilla and León Health Service-SACYL, Regional Health Management, 37005 Salamanca, Spain

[3] Emergency Service, University Hospital of La Paz P. of Castellana, 261, 28046 Madrid, Spain; letici.gomez@salud.madrid.org

[4] Research Network on Chronicity, Primary Care and Health Promotion (RICAPPS), 37005 Salamanca, Spain

[5] Home Hospitalization Service, Marqués of Valdecilla University Hospital, s/n, 39008 Santander, Spain; martagmzsnchz@gmail.com

[6] Department of Medicine, University of Salamanca, 28046 Salamanca, Spain

[7] Department of Biomedical and Diagnostic Sciences, University of Salamanca, 37007 Salamanca, Spain

* Correspondence: magomez@usal.es; Tel.: +34-923-291100-54757

† These authors participated in identical conditions as the first author of the manuscript.

‡ These authors participated in identical conditions as the last author of the manuscript.

Table S1. Characteristics of the Men analyzed by alcohol consumption.

	Non-drinker (n=36)		Low-moderate consumption (n=49)		Heavy consumption (n=12)		p value
	Mean	SD	Mean	SD	Mean	SD	
**Lifestyles**							
MD, (total score)	7.8	2.4	7.7	2.1	7.8	2.1	0.97
Time of smoker, (years)	24	12	25	13	26	11	0.88
METs-min/Week	5900	6100	4500	4400	7700	5000	0.14
**Conventional risk factors**							
Age, (years)	53	13	56	12	61	9.3	0.17
Evolution time, (months)	39	11	38	9.6	37	7.7	0.90
SBP, (mmHg) [b, c]	130	15	130	14	140	15	0.04
DBP, (mmHg) [b, c]	81	13	81	8.9	90	9.6	0.03
PP, (mmHg)	48	11	47	12	50	7.9	0.73
MAP, (mmHg) [b, c]	97	13	97	9.2	100	11	0.02
HR, (bpm)	71	15	71	12	76	15	0.57
FPG, (mg/dl)	96	26	91	14	100	18	0.23
Cholesterol total, (mg/dl)	180	36	190	29	180	39	0.51
LDL cholesterol, (mg/dl)	110	34	120	28	110	42	0.49
HDL cholesterol, (mg/dl)	47	11	49	11	53	11	0.21
Triglycerides, (mg/dl)	120	54	120	58	120	43	0.86
BMI, (kg/m^2)	30	5.5	29	4.3	31	3.2	0.44
Waist circumference, (cm)	100	15	100	11	110	9.8	0.48
**Estructura y función vascular**							
c-IMT, (mm)	0.66	0.10	0.68	0.12	0.73	0.13	0.16
cf-PWV, (m/s)	8.9	3.0	8.6	2.6	9.7	3.6	0.50
ba-PWV, (m/s)	13.6	2.6	13.4	2.3	14.8	2.1	0.18
CAVI	8.0	1.5	7.7	1.3	8.5	0.91	0.19

Values are means and standard deviations for continuous data. MD: Mediterranean Diet; MET: Metabolic Equivalent of Task; SBP: systolic blood pressure; DBP: diastolic blood pressure; PP: pulse pressure; MAP: mean arterial pressure; HR: heart rate; FPG: fasting plasma glucose; LDL-C: low-density lipoprotein cholesterol; HDL-C: high–density lipoprotein cholesterol; BMI: Body mass index; c-IMT: Intima–media thickness of common carotid; cf-PWV: carotid-femoral pulse wave velocity; ba-PWV: Brachial-ankle pulse wave velocity; CAVI: Cardio-ankle vascular index

p: statistically significant differences ($p < 0.05$).

Post hoc contrasts:

[a] Between 'non-drinker' and 'low-moderate consumption'.

[b] Between 'non-drinker' and 'heavy consumption'.

[c] Between 'low-moderate consumption' and 'heavy consumption'.

Rest of the groups $p > 0.05$.

Table S2. Characteristics of the Women analyzed by alcohol consumption.

	**Non-drinker (n=127)**		**Low-moderate consumption (n=72)**		**Heavy consumption (n=9)**		**p value**
	Mean	SD	Mean	SD	Mean	SD	
**Lifestyles**							
MD, (total score)	7.7	2.7	8.1	1.8	7.9	2.3	0.47
Time of smoker, (years) [b]	120	12	22	9.1	31	10	0.10
METs-min/Week	4700	4300	5600	5900	4800	4000	0.44
**Conventional risk factors**							
Age, (years)	50	12	53	9.8	54	12	0.13
Evolution time, (months) [a]	37	11	41	6.4	37	11	0.02
SBP, (mmHg)	120	17	110	14	120	16	0.17
DBP, (mmHg)	75	11	74	10	73	9.0	0.80
PP, (mmHg) [a]	43	10	39	7.4	42	7.9	0.03
MAP, (mmHg)	89	12	87	11	87	11	0.49
HR, (bpm) [b, c]	71	10	70	10	62	9.8	0.048
FPG, (mg/dl)	85	19	84	9.7	84	5.7	0.91
Cholesterol total, (mg/dl)	190	36	190	34	180	33	0.49
LDL cholesterol, (mg/dl)	110	31	120	29	100	29	0.40
HDL cholesterol, (mg/dl)	60	14	62	13	65	9	0.21
Triglycerides, (mg/dl)	98	52	92	40	72	17	0.22
BMI, (kg/m^2)	28	6.2	27	5.2	26	4.7	0.64
Waist circumference, (cm)	90	15	88	13	86	13	0.71
**Estructura y función vascular**							
c-IMT, (mm)	0.62	0.07	0.63	0.07	0.64	0.07	0.47
cf-PWV, (m/s)	7.1	1.8	7.0	1.8	7.9	1.9	0.34
ba-PWV, (m/s)	12.5	2.5	12.1	1.8	12.8	2.3	0.39
CAVI	7.2	1.7	7.4	1.1	7.8	0.8	0.22

Values are means and standard deviations for continuous data. MD: Mediterranean Diet; MET: Metabolic Equivalent of Task; SBP: systolic blood pressure; DBP: diastolic blood pressure; PP: pulse pressure; MAP: mean arterial pressure; HR: heart rate; FPG: fasting plasma glucose; LDL-C: low-density lipoprotein cholesterol; HDL-C: high–density lipoprotein cholesterol; BMI: Body mass index; c-IMT: Intima–media thickness of common carotid; cf-PWV: carotid-femoral pulse wave velocity; ba-PWV: Brachial-ankle pulse wave velocity; CAVI: Cardio-ankle vascular index

p: statistically significant differences ($p < 0.05$).

Post hoc contrasts:

[a] Between 'non-drinker' and 'low-moderate consumption'.

[b] Between 'non-drinker' and 'heavy consumption'.

[c] Between 'low-moderate consumption' and 'heavy consumption'.

Rest of the groups $p > 0.05$.
